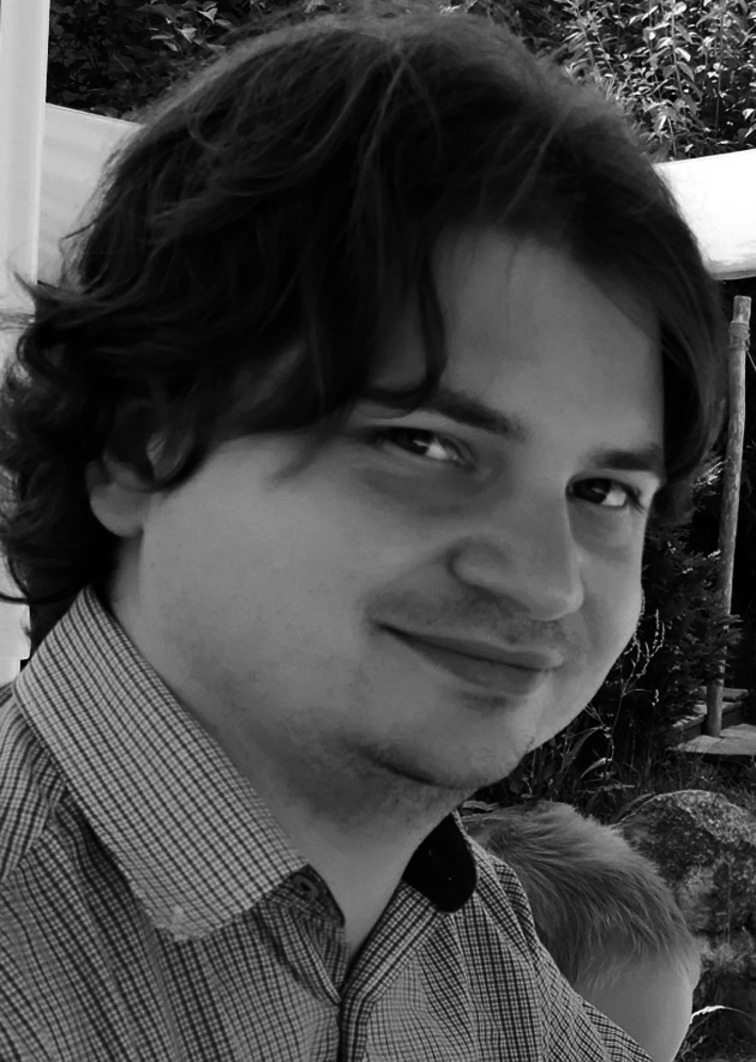# Hydrothermal Degradation of Amino Acids

**DOI:** 10.1002/cssc.202101487

**Published:** 2021-10-07

**Authors:** Paul Körner

**Affiliations:** ^1^ DBFZ - Deutsches Biomasseforschungszentrum gemeinnützige GmbH Biorefineries Department Torgauer Straße 116 04347 Leipzig Germany

**Keywords:** deamination, decarboxylation, high-pressure chemistry, hydrothermal carbonization, proteins

## Abstract

Within the past years, hydrothermal processes have gathered much attention as promising conversion technologies for especially wet biomass. Amino acids are an integral component of biomass, zoo biomass in particular. However, what happens to them during hydrothermal treatment? Reviewing the available literature going back to the mid of the 20th century revealed an astonishing, but still fragmentary view. In fact, two universal degradation reactions could be identified (i. e., deamination and decarboxylation), competing with each other. Thereby, small structural differences may obviously have huge impacts on the fate of individual amino acids. Nevertheless, the amount of available experimental data is relatively scarce in many cases. In this work, the available knowledge about the degradation of 20 proteinogenic amino acids under hydrothermal conditions was presented and discussed critically. The hydrothermal conversion of proteinaceus biomass as well as the Maillard reaction, both extensively reviewed elsewhere, were only touched on.

## Graphical abstract

**The missing part**: It is well known that hydrothermal processes can be used to convert biomass into solid, liquid, or even gaseous fuels. There are also innumerable scientific reports about the degradation of carbohydrates under hydrothermal conditions yielding especially furanic compounds. Fatty acids, in turn, can be expected to be rather inert during hydrothermal treatment. But what happens to another integral component of biomass: amino acids?

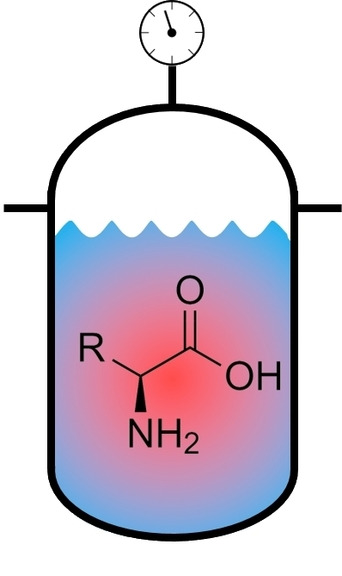

## 1 Introduction

Amino acids (AmA) are an essential brick of life on earth by building up peptides and proteins with manifold properties and tasks in living organisms. There are different possibilities to classify proteins: according to their function as either structural element or biocatalyst (i. e., enzyme); according to their outer shape as globular or fibrillary protein; according to whether and which other biomolecules, like sugars or lipids, are attached to them; and many more.^[1]^ All proteins have in common to be constituted of approximately 20 different α‐AmA merged by amide bonds between the α‐amino and the α‐carboxy group. Thus, like polysaccharides or fats, proteins are just another example of biogenic macromolecules being hydrolysable. This seems to be a crucial prerequisite for life as we know it: macromolecules, a structural material that can be decomposed simply be the action of water, another brick of life, but that does not readily decay in contact with water.

In hydrothermal processes (HTP) sub‐ or even supercritical water plays a crucial role, not just as solvent, but also as catalyst and reactant.^[2]^ While HTP are also a geological phenomenon this article will focus on their application for the thermal conversion of biomass. In this context, HTP bear the advantage that they can handle moist or even wet biomass. Thus, in most cases, the substrates can be used directly in different HTP as no drying is required. This is in contrast to other thermal conversion processes such as pyrolysis or dry gasification, which require dry or dried substrates. On the other hand, the high pressures in HTP generated by water are a challenge for reactor design and handling.^[3]^

In general, there are different types of HTP that can be distinguished by the operating temperature: hydrothermal carbonization (HTC) takes place in a range between 160 and 250 °C and mainly yields a lignite‐like solid that might be applicable as fuel or energy storage. By hydrothermal liquefaction (HTL), an oily biocrude is produced by the treatment of biomass between 300 and 350 °C, which can be further converted into biofuels. Lastly, from 350 °C hydrothermal gasification (HTG) becomes the dominating process, yielding a synthesis gas that consists of carbon dioxide, carbon monoxide, methane, and hydrogen.^[4]^

Overall, HTP are mainly studied for plant‐based biomass, which is of course related to the fact that plant‐based biomass is much more abundant than zoo biomass. Nevertheless, zoo biomass is also relevant. The main difference between plant and zoo biomass consists in the protein and, hence, AmA content. While there are also certain plants or components of plants being relatively rich in proteins zoo biomass is always rich in proteins. This is also one reason why the disposal of zoo waste is more problematic. Putrefaction does not only cause unpleasant odors but may also be a source of infectious diseases and an environmental threat.^[5]^

As an example, one remembers the mass culling of minks in Denmark in 2020 due to coronavirus mutations. Events like this one generate large amounts of zoo biomass in a short time. But also fishery waste is produced in significant tonnages throughout the world. Not least, residues of modern civilization, such as sewage sludge or digestate, even though not zoo biomass, contain proteins and AmA.^[6]^ In fact, HTP is particularly studied for the conversion of these residues. The handling of sewage sludge and digestate is increasingly challenging because of problematic constituents and a high load of nutrients.^[7]^ Therefore, many countries restrict the direct application as fertilizer and thus alternative conversion pathways are required.^[8]^ The purpose of HTP in this context is to convert these residues into products that are available for energetic or material use (e. g., char) and to recover nutrients like phosphorus.^[9]^ Also, the recovery of nitrogen (i. e., ammonium) is of interest, though it is less developed compared with strategies for phosphorus or phosphate recovery. A significant portion of the nitrogen contained in sewage sludge and digestate can be assumed to originate from AmA. In general, there are many residues with a high content of proteins and, thus, AmA. Therefore, distinct knowledge about the fate of AmA in HTP should be useful for the further development of hydrothermal conversion technologies.

Whenever large amounts of proteinaceus residues accrue that require hygienization, HTP may serve as an attractive alternative to burying or burning. It is also conceivable to generate valuable conversion products by the HTP of proteins or single AmA. Compounds like glutamic acid or ornithine can be relevant for a sustainable chemical industry.^[10]^

The purpose of this article is to review the existing knowledge about AmA degradation under hydrothermal conditions and, in doing so, to identify needs for further research.

## 2 Hydrothermal Degradation of Proteins and Peptides

Biomass rarely contains free AmA. They are usually bound in peptides and proteins. Prior to actual AmA degradation, therefore, hydrolysis takes place during hydrothermal treatment (HTT).[^11^, ^12^, ^13^]

In fact, this is comparable with monosaccharides obtained from polysaccharides prior to degradation. Ravber et al. investigated the fate of triglycerides, carbohydrates and proteins during HTT of sunflower seeds.^[14]^ They found that carbohydrates are the least stable component exhibiting significant degradation already at 130 °C. In contrast, triglycerides rarely hydrolyze at temperatures below 240 °C. Proteins are in between, showing a drastic hydrolysis from 190 °C. Ravber et al. explain these different behaviors by different interactions with subcritical water: carbohydrates swell, which increases the contact surface with water, while proteins coagulate, decreasing the contact with water, and triglycerides even form a second phase.

The coagulation of proteins at increased temperature certainly contributes to their enhanced hydrolysis stability; however, it is also a fact that amide bonds constituting the protein backbone are simply more stable than glycosidic bonds within polysaccharides. This can be seen with the polysaccharide chitin consisting of β‐1,4 glycosidic bonds, but bearing acetamide groups as well. An investigation by Einbu and Vårum on the chitin hydrolysis in hydrochloric acid at mild temperatures of 25–35 °C reveals that the depolymerization rate constant is larger than the deacetylation rate constant and increases much more strongly with increasing temperature and acid concentration.^[15]^

AmA generation from proteinaceus biomass via HTT has been studied for various examples as already well reviewed by Marcet et al.^[13]^ and Di Domenico Ziero et al.^[10]^ In this work, therefore, only a brief discussion shall be provided.

In the HTT of waste fish entrails using pure water an optimal AmA yield could be achieved at 250 °C within 60 min in a batch system. Applying lower temperature or reaction time resulted in an incomplete hydrolysis while at higher temperature or reaction time AmA degradation dominated.^[16]^ The same optimum was found for shrimp shells; however, only 25 % of the total AmA content could be recovered based on the acid hydrolysis.^[17]^

At 250 °C the ion product of water maximizes from which the claim arose that optimal AmA yields are always obtained at this temperature; however, other studies suggest that this is not reasonable. Of course, the protein hydrolysis profits from a high ion product as protons on the one hand act as catalyst to this reaction while hydroxide ions on the other hand serve as nucleophile substituting the amino group from the carbonyl carbon of the peptide bond. Nevertheless, the optimal AmA yield does not only depend on the protein hydrolysis rate but also on the AmA decomposition rate. In fact, AmA decomposition does not take place separately from hydrolysis. In a study with bovine serum albumin both hydrolysis rate constant and AmA decomposition rate constant were found to increase in a similar fashion between 250 and 330 °C, but the latter was always larger.^[18]^ In a study with bean dregs the AmA decomposition rate constant was lower than the hydrolysis rate constant at 200 °C, but at 240 °C it was larger than the hydrolysis rate constant while both constants were similar at 220 °C.^[19]^ Many studies report temperatures for optimal AmA yield far below, but also notably above 250 °C, such as red algae (120 °C),^[20]^ rice bran (200 °C),^[21]^ or bean dregs (330 °C).^[22]^ Thus, the conditions for optimal AmA yield do not only depend on a single solvent property but are also strongly influenced by the substrate and the process or experimental design.

Obviously, also the heating method strongly impacts the protein degradation as described by Chen et al. They demonstrated that under microwave irradiation protein hydrolysis in 6 n HCl usually taking 24 h by conventional heating gives the same AmA recoveries within a few minutes.^[12]^ Also with pure water as solvent (i. e., without catalyst), proteins are hydrolyzed much faster under microwave heating than under conventional heating, although the product profile is impacted as shown for silk protein.^[23]^ This is likely related to microwave irradiation not only heating up the solvent but also influencing the strength of covalent and non‐covalent bonds directly.

Overall, it can be stated that HTT leads to a rapid hydrolysis of proteins even in the absence of alkaline conditions or catalysts. However, it seems that protein hydrolysis under hydrothermal conditions cannot be decoupled from AmA decomposition completely. A work about the effect of cooking on beef proteins revealed that certain AmA modifications such as oxidation or pyroglutamate formation can already be found in the insoluble fraction, that is, prior to hydrolysis.^[24]^ Thus, understanding the degradation pathways of single AmA is a key to understanding the conversion of proteinaceus substrates under hydrothermal conditions.

## 3 Principles of Hydrothermal Amino Acid Degradation

Proteinogenic AmA bear at least one carboxy and one amino group, both at the same α‐carbon atom. Therefore, can be deduced that the abstraction of the amino group (i. e., deamination generating ammonia) and the abstraction of the carboxy group (i. e., decarboxylation generating CO_2_) are essential degradation pathways of α‐AmA. Especially from the fact that both functionalities are at the same atom it can be hypothesized that one AmA molecule might only undergo either decarboxylation or deamination, but not a sequence of both.

Of course, this is a simplification. If the reaction conditions are harsh enough, the whole molecule is degraded into ammonia and CO_2_, and, indeed, CO_2_ formation may be the definition for decarboxylation. However, in this work, decarboxylation is understood as the abstraction of a carboxy group rather than the conversion of originally non‐carboxy carbon into CO_2_. In this context, the work of Pietrucci et al.^[25]^ should be pointed out, who calculated the free energy of isovaline, an extra‐terrestrial AmA, and its hypothetic deamination and decarboxylation products. They showed that the product of sequential deamination and decarboxylation, 2‐butene, has a much higher free energy than the primary products, which are either deaminated or decarboxylated.^[25]^ Therefore, it is considered unlikely that a sequence of both degradation reactions may occur in one molecule.

An exception features the aromatic AmA. As detailed later, styrene can be observed as degradation product of phenylalanine, to name an example.^[26]^ In this case, the aromatic system is expanded by the generation of an additional double bond yielding a more stable product compared with an alkene.

Another question concerns the type of deamination reaction. For most AmA, both amino group elimination and amino group substitution products are reported. However, there seems to be no general rule as to which of these products are formed directly from the AmA and which are subsequent products. From alanine lactic acid is formed by amino group substitution that may undergo subsequent dehydration to acrylic acid.^[27]^ From aspartic acid maleic and fumaric acid are formed by amino group elimination and malic acid is a subsequent rehydration product.[^28^, ^29^] Elimination and substitution reactions usually compete. Thus, it should be expected that both amino group elimination and amino group substitution products can always form directly from the AmA and subsequently also interconvert into each other. However, which product is initially preferred differs and possibly depends on the availability of the β‐carbon atom for proton abstraction leading to elimination, and of the α‐carbon atom for water addition, respectively. Nevertheless, more research is necessary on this point. The same can be said about supposed reductive deamination products like propionic or succinic acid.

## 4 Degradation Pathways of Amino Acids under Hydrothermal Conditions

### 4.1 Glycine

**Scheme 1:**
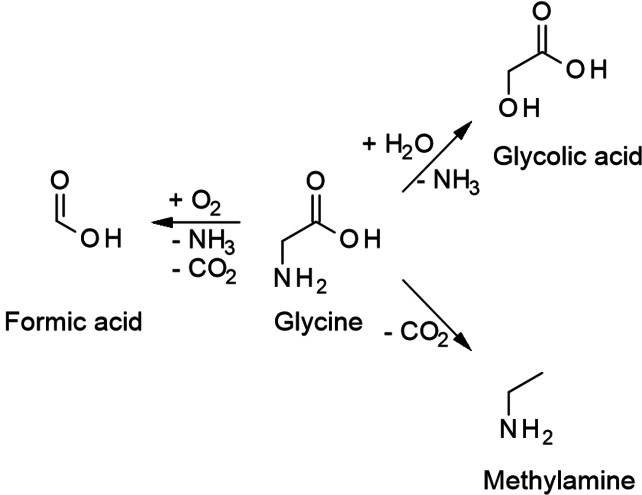
Degradation pathways of glycine.

As the simplest proteinogenic AmA, the reaction of glycine under hydrothermal conditions has been studied relatively extensively. The most prominent pathways are illustrated in Scheme 1. As two principal pathways, decarboxylation yielding methylamine and deamination are available. Deamination is a more complex reaction compared with decarboxylation, as several possibilities of amino group abstraction are conceivable. From glycine, glycolic acid is an observed deamination product, where the amino group is substituted by water or hydroxide, respectively.^[27]^ Amino group elimination, where a double bond is generated between α‐ and β‐carbon atom, is structurally impossible in the case of glycine (bearing no β‐carbon atom), but definitively observed in other AmA. Whether acetic acid may be considered as a plausible reductive deamination product of glycine is a matter of controversy as the amino group ought to be replaced by a hydride ion. The argument is that even if hydride or molecular hydrogen were present, they would rapidly undergo other reactions.^[25]^ Therefore, reductive deamination might be generally, not only for glycine, unfavorable, even though there are examples where saturated organic acids are found to be the product of unsaturated organic acids under hydrothermal conditions as detailed subsequently.

A final possibility for glycine deamination is coupled with oxidation and yields formic acid together with CO_2_.^[27]^

Unlike most other AmA, glycine can also be a degradation product, for example, of serine. Therefore, relatively high recovery rates are usually observed for glycine, which, however, are not related to an exceptional high stability, but to the fact that it is also formed during HTT.^[27]^

### 4.2 Alanine

Alanine is still quite similar to glycine as the residue is just one CH_3_ group instead of a hydrogen atom.

Decarboxylation leads to ethylamine, which might be further converted into ethanol by amino group substitution. Direct amino group substitution in alanine produces lactic acid, which may further dehydrate to acrylic acid. Acrylic acid can thus be considered as the amino group elimination product of alanine; however, the extent to which it is obtained from alanine directly and to which it is the product of lactic acid dehydration is not evident. Oxidative deamination of alanine yields pyruvic acid. Both pyruvic and acrylic acid are possibly subject to hydrogenation yielding lactic and propionic acid, respectively.^[27]^ Especially, the presence of propionic acid is difficult to explain without hydrogenation, which is still a doubtful reaction under these conditions as it requires a hydrogen source. Possibly, it makes a difference whether acrylic acid is hydrogenated or whether alanine undergoes a reductive deamination, even though both reactions yield the same product. In this context, hydrogen transfer reactions might play a role, such as the equilibrium between lactic and pyruvic acid.

Like glycine, alanine can be the degradation product of other AmA, especially aspartic acid, which is the reason for commonly high recovery rates.

### 4.3 Valine, leucine, isoleucine

**Scheme 2:**
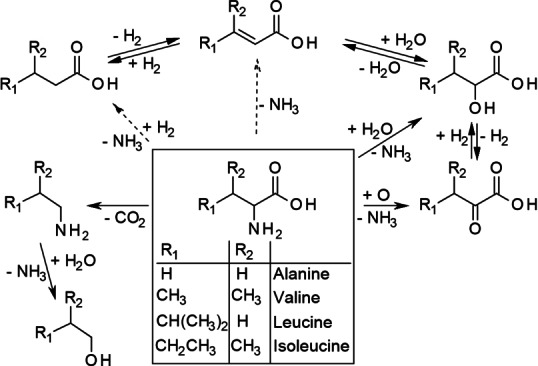
Decomposition pathways of alanine, valine, leucine, and isoleucine.

For valine, leucine, and isoleucine less information about the degradation under hydrothermal conditions is available. As they are structurally related with alanine, bearing longer but still unsubstituted alkyl chains, it can be assumed that they undergo analogous degradation reactions as displayed in Scheme 2.

Li and Brill, who studied the CO_2_ formation from AmA during hydrothermal treatment, found similar reactions rates for valine, leucine, and isoleucine at 320 °C, which are also within the same dimension as the reaction rate of alanine, supporting this hypothesis.^[30]^

A huge difference to alanine is that valine, leucine, and isoleucine are not expected to be produced from other AmA. Hence, the yield of these AmA is usually lower compared with alanine.^[31]^

### 4.4 Asparagine and aspartic acid, glutamine and glutamic acid

There are four structurally related AmA that despite exhibit very different behaviors during hydrothermal treatment. Aspartic and glutamic acid bear a terminal carboxy group in addition to the α‐carboxy group. Hence, it would be expected that both AmA degrade in a similar fashion and that the degradation pathway is mainly decarboxylation. Asparagine and glutamine can be considered as analogues to aspartic and glutamic acid, respectively, where the terminal carboxy group is replaced by an amide group.

**Scheme 3:**
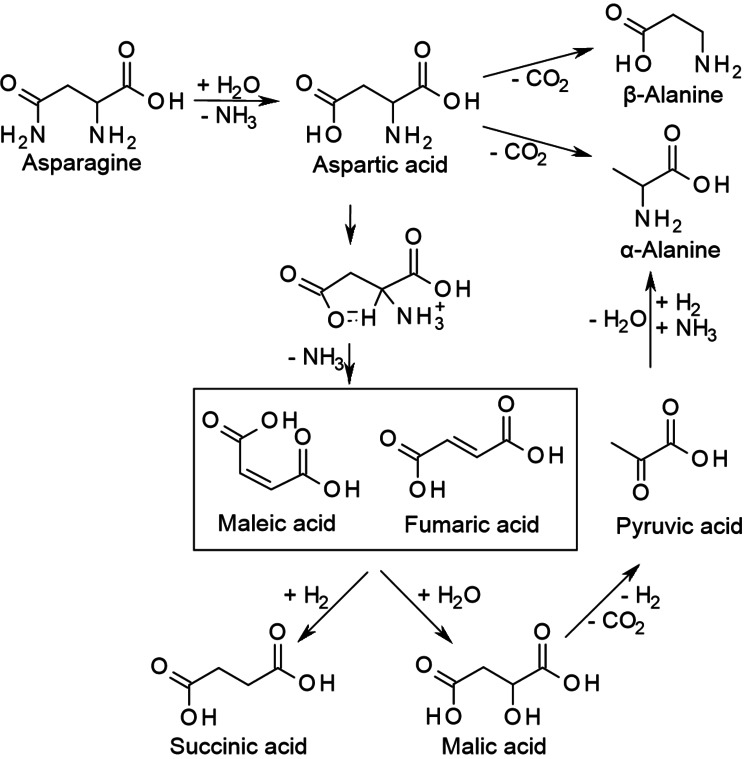
Decomposition pathways of asparagine and aspartic acid.

**Scheme 4:**
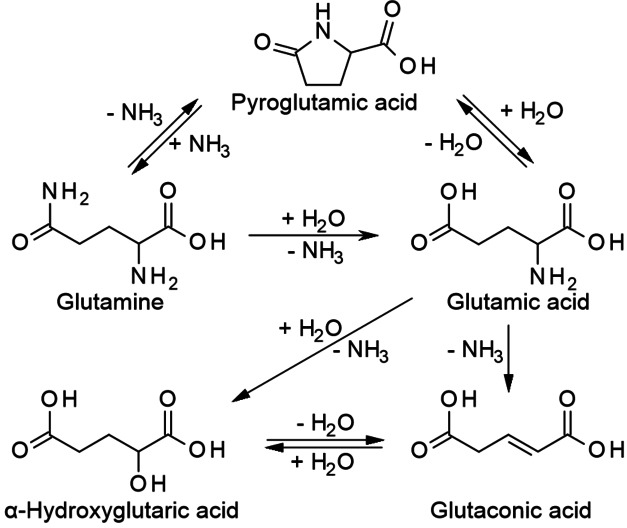
Decomposition pathways of glutamine and glutamic acid.

Indeed, all four AmA react very differently during HTC as illustrated in Scheme 3 and Scheme 4. For aspartic acid, deamination is found to be the main degradation pathway yielding primarily maleic and fumaric acid. Both are isomeric amino group elimination products. Their rehydration yields malic acid. In this case, literature suggests that malic acid is really the rehydration product of maleic and fumaric acid and not the direct amino group substitution product of aspartic acid as it is obtained in much lower yields.[^28^, ^29^] However, it might be possible that malic acid is an intermediate that is formed more slowly than it is consumed. Oxidative decarboxylation of malic acid leads to pyruvic acid, which may be further converted into acetic or lactic acid. Hydrogenation of maleic and fumaric acid gives succinic acid.^[28]^ Estrada et al. demonstrated that the addition of H_2_ strongly enhances the yield of succinic acid at the expense of malic, fumaric, and acetic acid.^[29]^ Decarboxylation of aspartic acid plays a minor role. Still, α‐ and β‐alanine can be obtained by the abstraction of the terminal or the α‐carboxy group, respectively, whereby α‐alanine dominates over β‐alanine.^[28]^ As another pathway to α‐alanine formation from aspartic acid the reductive amination of intermediate pyruvic acid is suggested.[^27^, ^29^] Beside alanine, also glycine was found to be a degradation product of aspartic acid. Estrada et al. assume that glycine might be formed from glyoxylic acid, a possible retro‐aldol product from malic acid.^[29]^

For the degradation of glutamic acid, the work of Sohn and Ho reports no ammonia generation while more than one ammonia molecule was generated per two aspartic acid molecules.^[32]^ In other works, glutaconic and α‐hydroxyglutaric acid have been identified as primary deamination products of glutamic acid being further converted into α‐ketoglutaric and succinic acid. However, the yields of ammonia did not exceed 1 mol%. In turn, pyroglutamic acid was formed with near‐100 % yield by the condensation of the amino group with the terminal carboxy group.^[33]^

Glutamine was found to produce nearly one molecule ammonia per molecule educt, suggesting that the amide group is either hydrolyzed into a carboxy group (giving glutamic acid) or undergoes condensation with the α‐amino group (giving pyroglutamic acid). Also, asparagine yielded much more ammonia than aspartic acid even though a covalent cyclization product is not favored in this case.^[32]^

From the reported observations for these four AmA it can be learnt that already a simple difference in the chain length of only one CH_2_ unit can have a huge impact on the degradation pathway. The β‐carboxy group in aspartic acid possibly catalyzes the abstraction of the α‐amino group via a five‐membered ring transition state. Therefore, deamination is the dominating degradation pathway.[^30^, ^34^] The same effect of the amide group in asparagine might be weaker. In glutamic acid and glutamine, however, cyclization does not lead to a transition state but to a stable product, inhibiting subsequent degradation. Possibly, pyroglutamic acid may act as a “storage form” from which glutamic acid or glutamine can be regenerated upon mild hydrolysis or ammonolysis, respectively.

### 4.5 Serine and threonine

Serine and threonine both bear a hydroxy group in their residues and are therefore expected to undergo dehydration reactions. In fact, dehydration of serine is postulated to yield aminoacrylic acid, which is in a tautomeric equilibrium with iminopropanoic acid. However, neither is observed but believed to be converted into pyruvic acid via amino group substitution.^[27]^ Decarboxylation of serine yields aminoethanol. In addition, glycine and glycine degradation products are observed from serine, leading to the assumption that serine undergoes retro‐aldol condensation to give glycine together with formaldehyde.^[27]^ However, a simple deamination of serine is not reported, likely because the abstraction of the hydroxy group is always favored over the abstraction of the amino group.

**Scheme 5:**
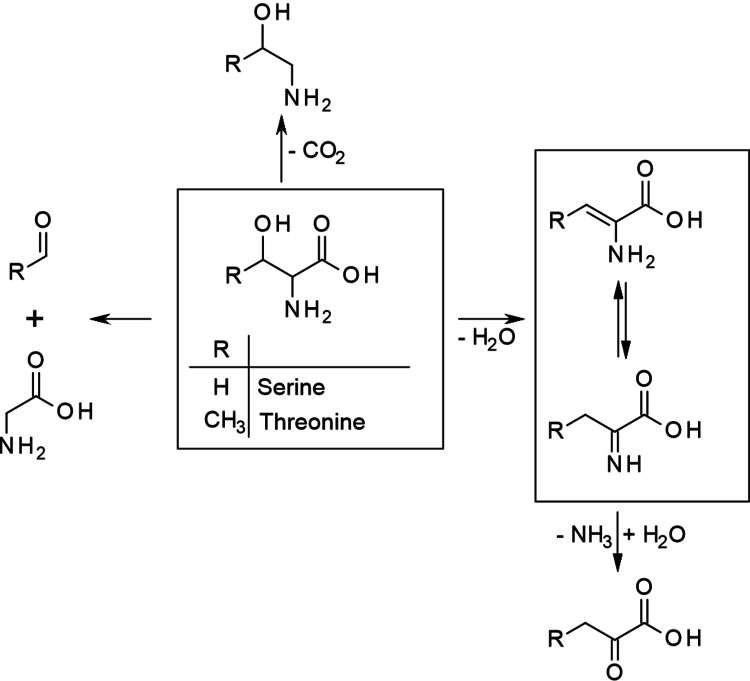
Decomposition pathways of serine and threonine

Threonine, which bears an additional methyl group, can in principle be expected to undergo the same reactions yielding analogous products as illustrated in Scheme 5, although, to the authors’ knowledge, no investigations on threonine decomposition products have been performed.

### 4.6 Proline

**Figure 1:**
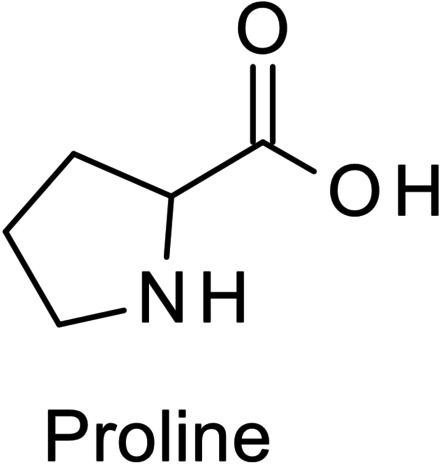
Structure of proline.

Proline is an unusual AmA as its amino group is not a primary amino group but part of a pyrrolidine ring as shown in Figure 1. Studies on the hydrothermal degradation of proline are rare. Tressl et al. reacted proline with different monosaccharides and identified several pyrrolidine derivatives in the reaction mixture, among them pyrrolidine itself, which is the decarboxylation product of proline. These results suggest that the pyrrolidine residue is quite stable; however, piperidine derivatives and especially condensation products of pyrrolidine and piperidine with carbohydrate degradation products were found as well.^[35]^

CO_2_ and ammonia formation during hydrothermal treatment of pure proline seems to be very low.[^30^, ^32^] Vallentyne found proline to be more stable than leucine, arginine, and lysine.^[36]^ Still, proline is rarely reported to exhibit a high recovery rate from proteinaceus biomass. Likely the most important consumption pathway of proline is not degradation but condensation with other compounds, rendering it a relevant precursor to nitrogen‐containing humins.

### 4.7 Methionine and cysteine

**Figure 2:**
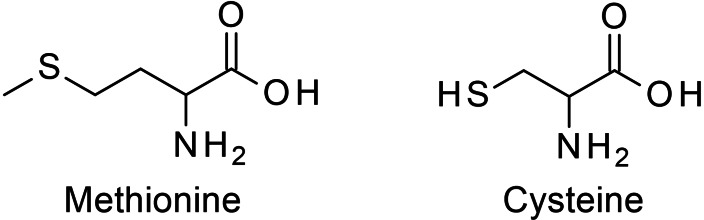
Structures of methionine and cysteine.

Methionine and cysteine are sulfur‐containing AmA. Compared with cysteine the alkyl chain of methionine is one CH_2_ unit longer and the thiol group is methylated as seen in Figure 2. Despite these small structural differences both AmA behave very differently during hydrothermal treatment. Cysteine is in principle the thiol analogue of serine; thus, the same types of degradation reactions should be expected (see Scheme 5). However, the ammonia generation from cysteine is much higher than from serine. In fact, deamination is the main degradation pathway of cysteine.^[32]^ Like for serine, deamination of cysteine should be coupled with a previous dehydration or, in this case, dethiolization yielding instable aminoacrylic and iminopropanoic acid that undergo subsequent deamination to pyruvic acid. That a thiol group is a better leaving group than a hydroxy group might serve as an explanation for the domination of this pathway over retro‐aldol condensation yielding glycine and thioformaldehyde and decarboxylation yielding cysteamine (2‐aminoethanthiol). An additional explanation is that the thiol group serves as nucleophile catalyzing the amino group abstraction; however, if this is true, cysteine should catalyze the deamination also of other AmA present in the solution.^[32]^

The ammonia generation from methionine is as low as for most other AmA.^[32]^ Likely, the abstraction of the methylmercapto group is less favored and would not initiate the deamination as there is an additional CH_2_ group between the methylmercapto group and the α‐carbon atom. There are findings suggesting an intramolecular interaction between the sulfur and the carboxy oxygen atom having a huge impact on the racemization rate of methionine and cysteine derivatives, that is, the cysteine derivative racemizes between 45 and 150 times faster than the corresponding methionine derivative.^[37]^ Nevertheless, it is open to question whether this allows any conclusions to the deamination or decarboxylation rate.

### 4.8 Lysine and arginine

Lysine and arginine bear additional nitrogen functional groups, namely a terminal amino and guanidine group, respectively. However, differently from what one would guess this does not make them good ammonia generators under hydrothermal conditions. From arginine the ammonia generation is slightly higher than from most other AmA, but still significantly lower than, for example, from aspartic acid. Lysine, however, is unremarkable in terms of ammonia generation.^[32]^

**Scheme 6:**
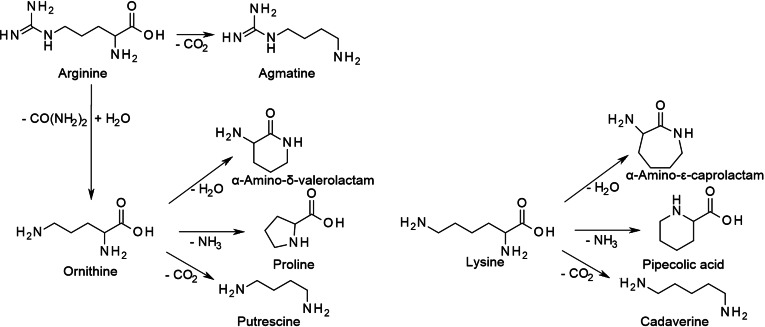
Decomposition pathways of arginine and lysine.

Li and Brill postulate that the guanidine group in arginine is hydrolyzed into a terminal amino group, that is, ornithine is produced, consequently together with urea as illustrated in Scheme 6.^[30]^ Urea is known to decompose quickly into CO_2_ and ammonia under hydrothermal conditions.^[38]^ Ornithine is assumed to form a six‐membered lactam by the condensation of the terminal amino group with the α‐carboxyl group.^[30]^ In 1968, Vallentyne identified ornithine as a product of hydrothermal arginine decomposition together with an unknown “post‐arginine”, possibly the mentioned lactam, and proline.^[36]^ The formation of proline would require a nucleophilic attack of the α‐amino group at the δ‐carbon atom substituting either the guanidine group in arginine or the amino group in ornithine. Thus, the proline formation, if it is true, should also produce ammonia as a side product.

Ornithine can, in principle, be considered as an analogue to lysine, which is just one CH_2_ unit longer. Hence, a lysine lactam would be possible but constitute a rather unfavorable seven‐membered ring. In turn, an intramolecular substitution of the terminal amino group by the α‐amino group would yield pipecolic acid as a six‐membered analogue to proline.

Decarboxylation of arginine should, under structural considerations, yield agmatine (4‐aminobutylguanidine). Ornithine and lysine would give putrescine (1,4‐diaminobutane) and cadaverine (1,5‐diaminopentane), respectively. Especially, the latter two are well known products of microbial protein degradation responsible for the characteristic smell of putrefying bodies.^[39]^

### 4.9 Aromatic amino acids

**Scheme 7:**
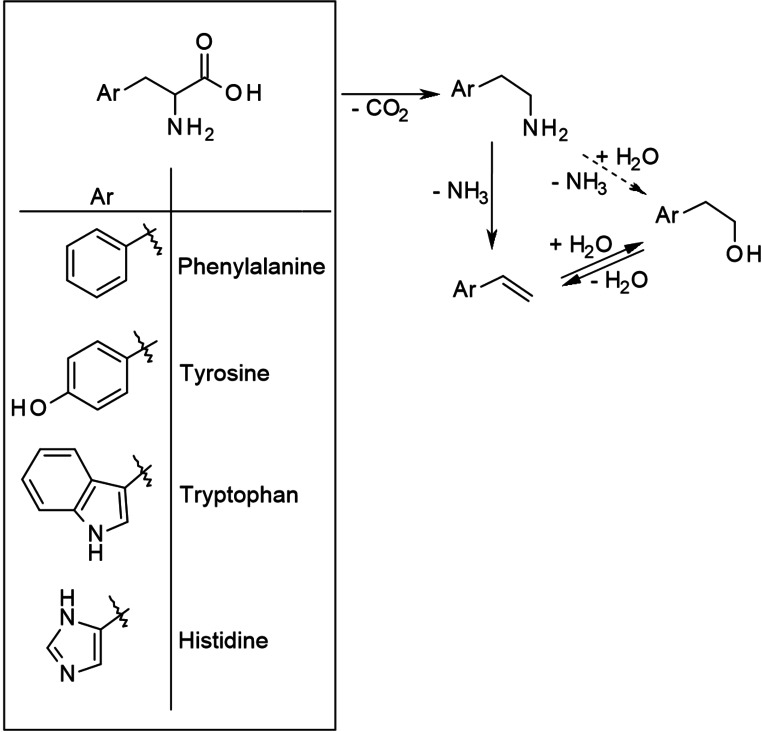
Decomposition pathways of aromatic AmA.

The aromatic AmA phenylalanine, tyrosine, tryptophan, and histidine share a common degradation pathway despite comparably large structural differences. First of all, as the residue is aromatic, it is very resistant. As an example, Samanmulya et al. demonstrated that the imidazole ring of histidine even survives hydrothermal gasification as 4‐methylimidazole was obtained in yields up to 80 mol%.^[40]^ Thus, the main degradation takes place around the α‐carbon atom as illustrated in Scheme 7.

This is well investigated for phenylalanine. In this case, decarboxylation leads to phenylethylamine as a major decomposition product. However, subsequent amino group elimination yielding styrene is also possible. A sequence of α‐decarboxylation and α‐deamination is usually unexpected. However, in the case of aromatic AmA the conjugated system is expanded by the generation of an additional double bond yielding a relatively stable product. Styrene subsequently undergoes rehydration to 1‐phenylethanol and 2‐phenylethanol.^[26]^ In principle, these products can also be considered as the amino group substitution products of phenylethylamine and as the precursors of styrene via dehydration, although this is not clear. Obviously, they are obtained in a lower yield than styrene.^[26]^

Not reported is the possibility that deamination takes place prior to decarboxylation, which would yield cinnamic acid or phenyllactic acid as styrene precursors. Possibly, deamination is generally less favored as in all strong ammonia generators identified by Sohn and Ho ammonia is either released from the residue or certain functional groups of the residue act as deamination catalyst.^[32]^ However, neither effect is apparent for the aromatic AmA.

**Scheme 8:**
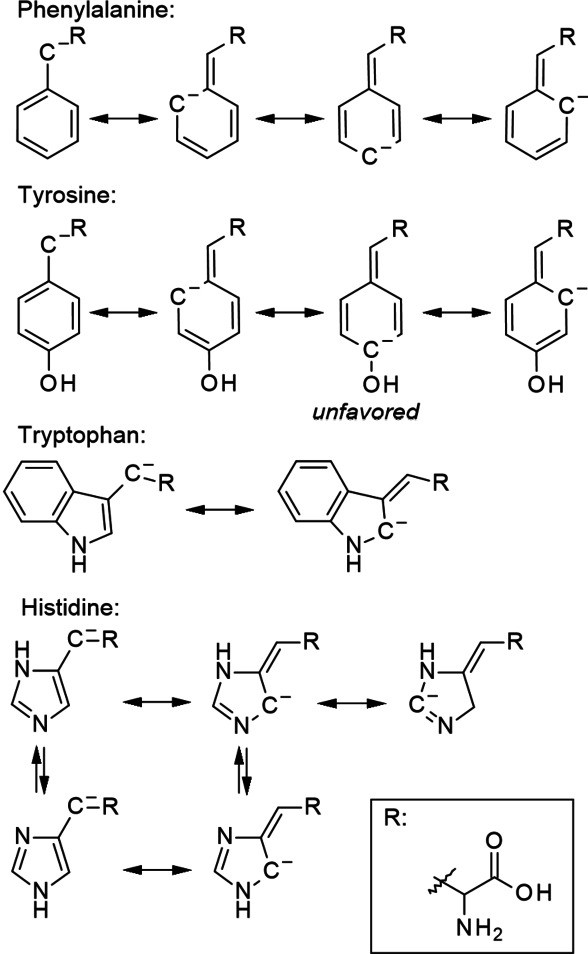
Resonance structures of aromatic AmA β‐carbanion. The resonance structures of tyrosine β‐carbanion are not favored because of the competing +M effect of the phenol group.

An interesting observation is made already in 1969 by Bryan and Olafsson.^[41]^ They demonstrated that phenylalanine decomposes at the lowest temperature (among the aromatic AmA) followed by histidine, tryptophan, and, with a difference of around 40 K to phenylalanine, tyrosine, even though tyrosine (i. e., 4‐hydroxyphenylalanine) is structurally closely related with phenylalanine. This succession can be explained by different mesomeric effects of the residues. Assuming that the deamination of aromatic AmA follows an E1cb mechanism, then first a deprotonation of the β‐carbon atom takes place generating a carbanion. This carbanion is stabilized by the aromatic residue via resonance, however, to different extent as illustrated in Scheme 8. In phenylalanine, the stabilization is the strongest even though a phenyl group is already electron‐releasing. The stabilization by the unprotonated imidazole ring and the indole ring of histidine and tryptophan, respectively, is weaker as first the electron‐releasing (i. e., +M) effect is stronger and, second, at least for tryptophan, fewer resonance structures exist. In the case of tyrosine, the stabilization is the weakest because of a strong +M effect caused by the phenol group.

Nevertheless, this hypothesis is vulnerable as the main decomposition pathway of aromatic AmA is obviously not amino group elimination, but decarboxylation. According to another work of Li and Brill, the decarboxylation of AmA is catalyzed by water, that is, one water molecule transfers a proton from the α‐amino group to the α‐carbon atom while the bond to the carboxylate group is cleaved releasing CO_2_.^[42]^ Possibly, a higher electron density at the β‐carbon atom has a diffuse inhibiting effect on the cleavage of the bond between α‐ and carboxylate carbon atom. Another possibility would be that water does not only act as “proton pipeline” between the amino group and the α‐carbon atom, but also between the β‐carbon atom and the α‐carbon atom. In this case, a high electron density at the β‐carbon atom would impede the proton transfer to the α‐carbon atom.

### 4.10 Dimerization and Oligomerization

**Scheme 9:**
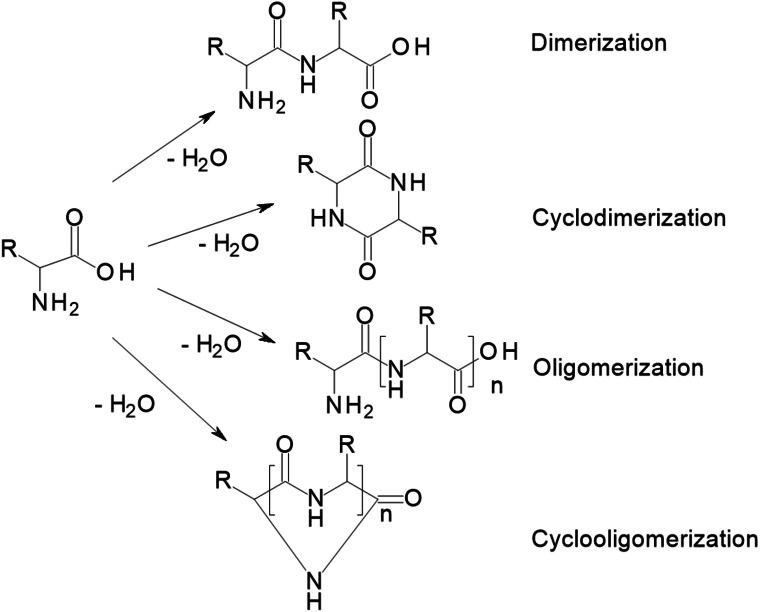
Possible dimerization and oligomerization reactions of AmA.

Dimerization and oligomerization are commonly observed reactions of AmA. Those were especially studied for glycine and alanine, but it can be assumed that all AmA may undergo such reactions, maybe to different extent. For glycine and alanine, up to pentamers are reported together with so called diketopiperazines, that is, double‐condensed dimers as displayed in Scheme 9.^[43]^

Generally, it is conceivable that also residual amino or carboxy groups can be involved in oligocondensation reactions. Thus, HTT of proteins does not only destroy them, it can also reconstruct them and incorporate decomposition products of AmA and other molecules.

A captivating kind of AmA dimerization is based on the formation of dehydroalanine or dehydrohomoalanine, respectively. This reaction is basically described as the cause of intra‐ and intermolecular protein cross‐linking. Hence, it takes place long before protein hydrolysis with manifold effects on the protein's properties, such as stainability or digestibility. An extensive Review on this reaction and its significance is provided by Friedman.^[44]^ This work will give only a brief discussion concerning its implications for the fate of AmA under hydrothermal conditions.

**Scheme 10:**
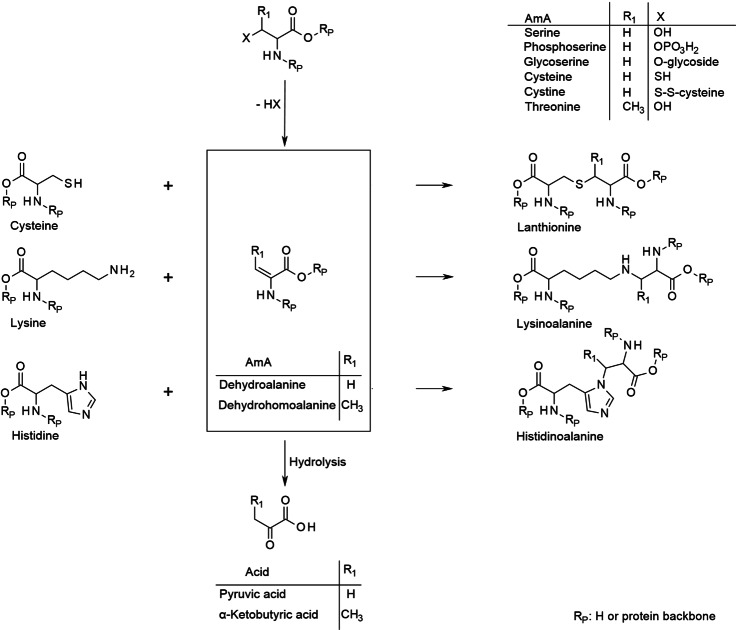
AmA dimerization based on the formation of dehydroalanine or dehydrohomoalanine, respectively.

As illustrated in Scheme 10, the β‐carbon functionality of serine, cysteine, and their derivatives can suffer elimination yielding dehydroalanine (i. e., aminoacrylic acid to maintain the terminology). The same may happen with threonine giving dehydrohomoalanine. These dehydro compounds are targets of nucleophilic attacks especially by amino or thiol groups. As the elimination takes place in still intact proteins, particularly lysine, histidine, and cysteine bearing suitable functional groups in their residues serve as attackers yielding lysinoalanine, histidinoalanine, and lanthionine residues (or their methyl analogues), respectively, that cross‐link the protein. However, other cross‐linking AmA such as ornithinoalanine or phenylethylaminoalanine are also described.^[44]^

Upon complete protein hydrolysis these products may be released, if not hydrolyzed before. In fact, the rehydration of dehydroalanine or hydrolysis of its addition products yields racemic serine (analogously threonine from methyldehydroalanine). The reaction with ammonia gives β‐aminoalanine (or β‐aminohomoalanine). Further degradation ends up in pyruvic acid (or α‐ketobutyric acid).^[44]^

Nevertheless, it is also conceivable that this type of reaction proceeds between free AmA or may even involve AmA degradation products. In this case, the variety of nucleophiles is greater as especially the amino groups are no longer bound in peptide bonds. Hence, a greater variety of products should be expected, each in a lower concentration though.

Overall, all kinds of dimerization and oligomerization are always reversible reactions that consume AmA, but form intermediates from which AmA can be yielded back prior to entering irreversible degradation.

### 4.11 Maillard Reaction

The Maillard reaction (MR) essentially is the condensation of a reducing sugar with an amino compound, especially AmA. Successive rearrangement, enolization, and fission reactions lead to variety of reactive intermediates that undergo subsequent reactions with additional amino compounds or other intermediates. Finally, polymeric molecules, so called melanoidins, form.[^45^, ^46^] To elucidate the entire complex reaction network that is still not understood in detail would go beyond the scope of this work. A comprehensive and still topical Review on the MR is provided by Hodge.^[45]^

Overall, the MR is not specific for AmA but for amines, of which AmA form only a subgroup. However, as elucidated in former sections of this work, amines are substantial degradation products of AmA. They are involved in the sugar–amine condensation, which can be considered as the initial step of the MR, preceding the Amadori rearrangement. They also undergo condensation reactions with various carbonyl compounds formed within other pathways of the MR to yield aldimines and ketimines at first, which subsequently polymerize to melanoidins. Amines might even react with carbonyl functional groups of already precipitated polymers causing the incorporation of nitrogen. In any case, melanoidins constitute an end‐product of the MR.^[45]^

**Scheme 11:**
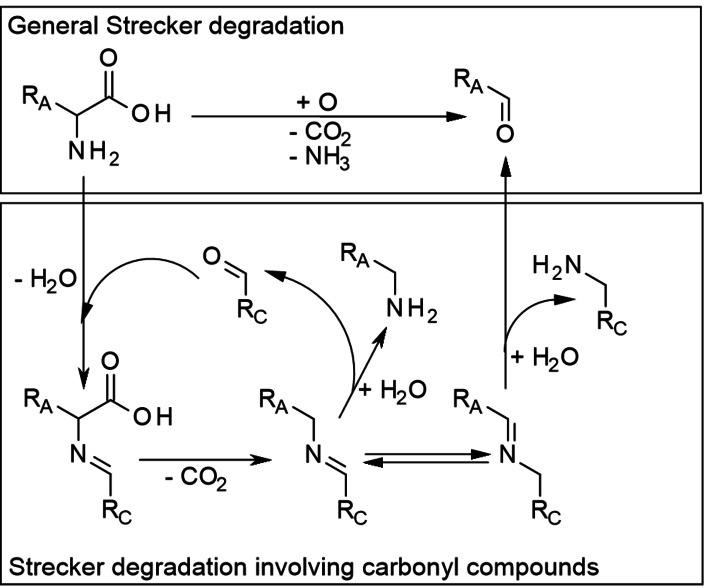
Strecker degradation of AmA generally and involving carbonyl compounds. It has to be noted that not all carbonyl compounds can participate in the SD. Usually, certain other α‐functionalities have to be present as well. In this regard the depiction is simplified.

One pathway of the MR being indeed specific for AmA is the Strecker degradation (SD). Generally, the SD comprises the oxidation of an AmA to an α‐iminoacid followed by its decarboxylation and hydrolysis yielding the so called Strecker aldehyde as illustrated in Scheme 11. The SD of AmA was already comprehensively reviewed by Schönberg and Moubacher^[47]^ and in a more recent work by Rizzi.^[48]^ Therefore, only the most relevant aspects in the context of hydrothermal AmA degradation are reported here.

In fact, certain types of carbonyl compounds, especially α‐dicarbonyls, act as oxidant in the SD, although numerous other oxidants have been identified, among them even inorganic compounds like ozone or hydrogen peroxide.^[47]^ However, those do not play a significant role in the thermal treatment of pristine biomass. In turn, α‐dicarbonyls originate from the dehydration of carbohydrates (e. g., to desoxyosones) or their fragmentation (e. g., to glyoxal or diacetyl). In this case, the initial imine formation consists in the condensation between the AmA and the carbonyl compound, which is followed by decarboxylation and hydrolysis. Depending on where the nucleophilic attack of a water molecule happens during the hydrolysis step, either the Strecker aldehyde is formed together with an amino compound, that is, a transamination takes place, or the nitrogen atom persists at the AmA residue, and the original carbonyl compound is yielded back. The latter case, where the carbonyl compound essentially acts as decarboxylation catalyst to the AmA, seems to be rather rare. On the other hand, α‐aminocarbonyl compounds formed from α‐dicarbonyls in the former case are very reactive and enter polymerization or further hydrolysis quickly; for example, instead of aminoacetone (from pyruvaldehyde) corresponding pyrazine derivatives and acyloins are observed.[^48^, ^49^]

Beside α‐aminocarbonyls, Rizzi describes α‐olefinic carbonyls (e. g., furfurals, α‐cyclopropylcarbonyls, α‐epoxycarbonyls, α‐epoxyenals, α‐epoxyenones, and 4‐hydroxy‐2‐alkenals), some of which are products upon lipid degradation, as undergoing “Strecker‐like reactions” and quinones, derived especially from polyphenols, and Amadori compounds as reactants in Strecker aldehyde formation.^[48]^ Thus, a wide range of compounds formed in the (hydro)thermal treatment of biomass is obviously able to induce SD of AmA. The products of SD in turn may enter various pathways of the MR or even participate in the SD again.

After all, this section clarifies that the presence of other organic compounds, especially those being or forming carbonyls, offers additional reaction pathways for the consumption of AmA and may, hence, strongly affect the fate of AmA under hydrothermal conditions.

## 5 Conclusion

Amino acids (AmA) are an integral component of biomass, zoo biomass in particular. However, they rarely occur as free AmA, but are condensed to proteins, which differ strongly in their physical and chemical properties. During hydrothermal treatment (HTT), proteins are hydrolyzed into single AmA. Nevertheless, AmA degradation even starts prior to protein hydrolysis. Hence, it is impossible to decouple protein hydrolysis and AmA degradation completely.

When focusing on the fate of single AmA during HTT two universal degradation reactions can be identified, that is, deamination and decarboxylation. In most cases, an indirect competition between those two degradation reactions has to be stated. The simple reason is that a sequence of both reactions in a single molecule is energetically unfavorable. An exception features aromatic AmA, where a sequence of both reactions expands the conjugated system, yielding a certain stability.

Interestingly, deamination dominates only the degradation of some AmA while most AmA undergo mainly decarboxylation. However, small structural differences may have huge impacts on the individual fate as reported, for example, for glutamic acid and aspartic acid.

For many AmA, the amount of available experimental data is relatively scarce, such as for proline or threonine. Thus, more research is required.

The effect of accompanying other organic molecules on the AmA degradation cannot be overestimated. In HTT, usually biomass featuring a complex mixture of organic molecules is converted. Under these circumstances, AmA dimerize or oligomerize with other AmA being present. Even more important is the condensation of AmA or their degradation products with carbonyl compounds, especially carbohydrates or molecules obtained from the carbohydrate conversion. This complex of pathways known as Maillard reaction yields insoluble, nitrogenous polymers called melanoidines that feature one end‐product of the HTT of proteinaceus biomass.

As many biogenic residues that are common or possible substrates for hydrothermal processes (HTP) contain AmA in significant amounts, it is astonishing to recognize the deficient knowledge about AmA behavior during HTT. Systematic studies concerning the effect of reaction temperature, time, pH value, and metal ions on the decomposition of different AmA are required as a basis for understanding the fate of proteins and proteinaceus substrates during HTT. It is hoped that this Minireview may support the filling of these knowledge gaps.

## Conflict of interest

The authors declare no conflict of interest.

## Biographical Information

*Dr. Paul Körner received his Master degree in Chemistry from University of Technology Dresden, Germany, in 2015 and his Ph.D. from University of Hohenheim, Germany, in 2020. At present, he is working at DBFZ ‐ Deutsches Biomasseforschungszentrum gemeinnützige GmbH, Germany. His research focusses on hydrothermal treatment of biogenic residues for the production of platform chemicals and recovery of nutrients*.